# Description of a Portable Wireless Device for High-Frequency Body Temperature Acquisition and Analysis

**DOI:** 10.3390/s91007648

**Published:** 2009-09-28

**Authors:** David Cuesta-Frau, Manuel Varela, Mateo Aboy, Pau Miró-Martínez

**Affiliations:** 1 Technological Institute of Informatics, Polytechnic University of Valencia, Alcoi Campus, Plaza Ferrandiz y Carbonell, 2, 03801, Alcoi, Spain; 2 Department of Internal Medicine, Mostoles Hospital, Madrid, Spain; E-Mail: mvarela.hmtl@salud.madrid.org; 3 Department of Electrical Engineering, Oregon Institute of Technology, Oregon, USA; E-Mail: mateoaboy@mac.com; 4 Department of Statistics, Polytechnic University of Valencia, Alcoi Campus, Alcoi, Spain; E-Mail: pamimar@eoi.upv.es

**Keywords:** body temperature, portable medical devices, wireless data transmission, approximate entropy

## Abstract

We describe a device for dual channel body temperature monitoring. The device can operate as a real time monitor or as a data logger, and has Bluetooth capabilities to enable for wireless data download to the computer used for data analysis. The proposed device is capable of sampling temperature at a rate of 1 sample per minute with a resolution of 0.01 °C . The internal memory allows for stand-alone data logging of up to 10 days. The device has a battery life of 50 hours in continuous real-time mode. In addition to describing the proposed device in detail, we report the results of a statistical analysis conducted to assess its accuracy and reproducibility.

## 1. Introduction

Temperature is a continuous variable that can be accurately measured and analyzed quantitatively. However, in clinical practice it is primarily used as an intermittent qualitative variable with often a poorly defined cut-off point. Despite its potential as a noninvasive physiologic signal to continuously assess patient status, its clinical use is often limited to a simple binary decision. While research studies have shown the usefulness of the temperature time-series for patient assessment and its prognostic value has been documented [1–3], the use of high-frequency/high-accuracy temperature time-series and associated analysis metrics have not been extended to clinical environments due to the lack of commercial temperature monitoring systems with adequate sampling frequency, resolution, and analysis capabilities. Consequently, even though thermal physiology has undergone big conceptual and technical changes, clinical thermometry has remained a rather stagnant discipline, and its conceptual framework has remained static for the last decades due to the lack of adequate monitoring systems.

Recent work involving analysis of high-accuracy and high-frequency temperature recordings has shown the importance of analyzing temperature as a dynamic time-series as opposed to a simple constant only useful to make a simple binary decision (febrile vs. nonfebrile). These important findings and clinical applications require medical devices capable of monitoring high-resolution temperature at a rate of 1 sample per minute during a period of several days. For instance, analysis metrics derived from the temperature time-series have been shown to correlate with the clinical status in critically ill patients [3]. Specifically, analysis metrics derived from the temperature time-series have been shown to serve as a good indicator of poor prognosis in patients with multiple organ failure. In this study it was shown that the predictive ability of the metrics derived from the temperature-time series alone were comparable to the Sequential Organ Failure Assessment (SOFA) score. A different study illustrated how analysis of the temperature-time series provides clinically useful information for noninvasive and real-time status assessment, since the metrics derived from the temperature time-series have been found to correlate with patient survival [1, 2].

In this paper we describe a device for dual channel body temperature monitoring—the TherCom monitor. The device can operate as a real time monitor or as a data logger, and has Bluetooth capabilities to enable for wireless data download to the computer used for data analysis. In several areas, recording the evolution of a clinical variable through time, instead of taking an isolated reading, has greatly improved its utility and has resulted in important clinical consequences. Examples could be Holter ECG monitoring [4], ambulatory monitoring of arterial pressure or continuous pulsioximetry [5].

The proposed device is capable of sampling temperature at a rate of 1 sample per minute with a resolution of 0.01°C. The internal memory has capability for up to 10 days of stand-alone data logging. The device has a battery life of 50 hours in continuous real-time mode. In addition to describing the proposed device in detail, we report the results of a statistical analysis conducted to assess its accuracy and reproducibility.

The temperature device we propose in this paper has been designed to capture the dynamic aspects of temperature in order to enable researchers to conduct further studies in the area of temperature regulation, thermal physiology, and clinical thermometry. Additionally, it is intended to serve as a clinical device to enable physicians to implement current advances in thermal physiology as part of their day-to-day clinical practice. Specifically, in addition to serve as a research tool, the proposed device has been designed to address common problems encountered in the clinical practice. These include:

- Monitoring temperature in admitted patients. Temperature is normally measured every 8 to 12 hours. Common knowledge assumes that if the patient develops a fever, either it will persist until the next lecture, or the patient will be so sick to call the nurse, and the fever will be detected. Nevertheless, there are no empirical studies to back up this practice. A continuous monitoring of the patient's temperature can help detect intermittent fever bouts and unleash the consequent diagnostic and therapeutic interventions more objectively.
- Detecting fever in an outpatient setting. A common complaint in an outpatient clinic is the presence of fever. Fever often develops in the evening, and may not be confirmed during the visit to the clinic. Having the capability to record for a 24 or 48 hour period (“temperature Holter monitoring”) eliminates this problem and enables clinicians to arrive to more precise diagnosis.
- Detecting bacteremia as early as possible, eventually before the fever spike. The presence of bacteria in blood triggers the release of a number of cytokines that stimulate certain hypothalamic nucleii and start several physiologic measures aiming to rise the core temperature. The first of these mechanisms is a vasoconstriction of peripheral vessels, aiming to bypass the blood from the subcutaneous tissue to the deep tissues and therefore minimizing heat dissipation. This is reflected in an abrupt fall in peripheral temperature. Measuring simultaneously central and peripheral temperature has the potential to provide early signs of the development of bacteremia prior to the appearance of a full-blown fever. Furthermore, blood cultures would arguably be more sensitive in this setting than once the fever mechanisms have been unleashed.
- Providing new ways of categorizing fever. Classic fever patterns (i.e., “hectic”, “recurrent”, etc) are now considered obsolete by the research community, and are seldom used in clinical practice. New analytical techniques derived from complex time-series analysis (approximate entropy [6, 7], sample entropy [8], Lempel Ziv algorithm [9, 10], detrended fluctuation analysis [11]) have been shown useful to study and classify temperature, even in the absence of fever. For instance, it has been shown that there is a good correlation between physiological status and ApEn of the temperature profile in critically ill patients, and that ApEn is a prognostic marker as good as conventional scoring systems, with the advantage of being non-invasive and a continuous, rather than episodic, evaluation [12].

Similar devices have been reported in the technical literature [13–18], or are even commercially available [19–21]. However, none of them includes simultaneously all the features of the device described in this paper:

- Two functioning modes: Holter mode for long term ambulatory recordings, and real time mode.
- Real time wireless data transmission. Some devices need a cable (RS232, USB) to download the data.
- Standard Bluetooth radio interface. Other radio interfaces employed are not standard and therefore specific modules are necessary. Additionally, Bluetooth can be used directly in many mobile devices such as PDAs and phones.
- Enables patient movement. Even for admitted critically ill patients, some patient movement is necessary (surgery, X-Ray). Wireless data transmission can be resumed without data loss (internal buffer of up to 10 days) when wireless communication is out of range.
- Long term ambulatory monitoring. Only generic data loggers enable long term monitoring, not specific for medical applications [19, 20].
- Lightweight and small size. Portable device in contrast to other in-site monitors [21].
- High accuracy: 0.2°C. The accuracy found in other devices is usually lower [19]. Additionally, calibration with proper resistors can increase the accuracy, up to a threshold set by the probe accuracy.
- Signal processing based on signal entropy. Usually manufacturers only provide data editors as software tools.
- Two input channels. Comparative temperature analysis can be carried out.
- Non-invasive, comfortable. It can be applied to healthy subjects as control subjects, not only pathological subjects.
- Hot battery recharging. Batteries can be recharged by means of a mini USB connector, while in operation. This can increase the actual battery life indefinitely.
- High frequency. Readings are taken every minute, by averaging 60 measurements, one per second.
- Additionally, this device has enabled researchers to obtain important clinical results, as described in [12].

## 2. Device Description

**Figure 1.**
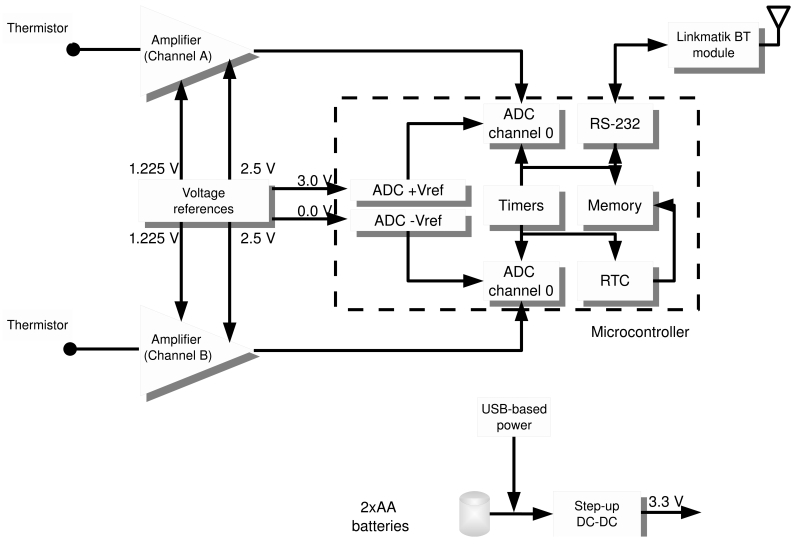
Block diagram of the high-accuracy/high-frequency temperature monitoring device (TherCom).

This section describes the main components of the device. Figure 1 shows a block diagram of the TherCom device and how the components are interrelated. The temperature monitor includes temperature sensors, a signal conditioner and amplifier for each channel, an analog to digital converter, a microcontroller, flash memory, and a Bluetooth module. It also includes a DC-DC converter to power the device from two standard AA batteries. Samples are transmitted via Bluetooth connection to the host computer at a rate of one sample per minute in real time mode, or off-line in data logger mode. At the host computer, data can be visualized and processed. The software tool includes a number of menu options for signal editing, entropy calculations, time-series analysis, and device configuration.

Each block is described in detail in the following subsections in order to ensure reproducibility by other members of the research community interested in implementing their own system.

### 2.1. Temperature Sensors

We selected YSI400 thermistors as the temperature sensors. The accuracy of the sensors is *±*0.02 °C and the resolution is 0.01 °C. Thermistor readings were converted using the standard YSI400 curve and the Steinhart-Hart polynomial which provides an accuracy far better than that of the sensors or parts themselves, *±*0.0025 °C at 25 °C with a resolution of 0.0001 °C. To calibrate the device, individual ultra high precision resistors (0.01% tolerance, *±*2 ppm/°C) were connected in place of the thermistors. We adjusted the gain and offset simultaneously by means of linear interpolation between reference points obtained.

As shown in the figure, the sensor resistance is turned into voltage by means of a precision current source, and this voltage then enters the amplifier to get an output range from 0 V to 3.0 V. The temperature measurements range from 31.00°C to 41.00°C. Self heating is avoided by powering the sensors during a very short period of time. Sensors can be attached to the skin of the patient for peripheral temperature acquisition, or be embedded in an orifice (usually the ear canal) for central temperature monitoring.

### 2.2. Thermistor Amplifier

**Figure 2.**
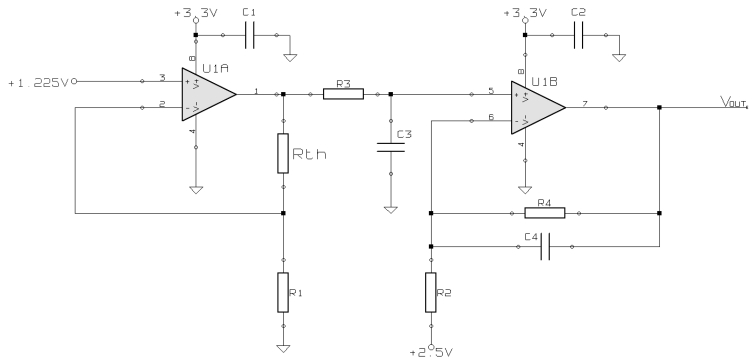
Schematic of the thermistor amplifier circuit. The first opamp circuit plays the role of a precision current source to feed the thermistor, and the second one is the amplifier itself.

To obtain the temperature from a thermistor it is necessary to measure its resistance by quantifying the voltage across it. This is carried out by the thermistor amplifier shown in Figure 2.

The first stage of this circuit is a precision current source. As current intensity varies along with the thermistor resistance, it has to be kept constant. This is accomplished by *U*_1_*_A_* configured as a current source. Current delivered to the thermistor *R_th_* is given by the input voltage and *R*_1_ using Ohm's law. To ensure high accuracy, input voltage is obtained from a precision reference, and *R*_1_ must be a low tolerance resistor (0.1 %). Since the minimum thermistor resistance is 1.152 Ω at 41.00 °C, and the maximum is 1.739 *Omega;* at 31.00 °C [22], we chose an input reference voltage of 1.225 V (see Section 2.4.) and *R*_1_ *=* 1*K*58. Thus, the current flowing through *R_th_* is constant and equals 77.5 mA.

The output *R-th* voltage is low-pass filtered by *R_3_* and *C*_3_. The values chosen for these parts are 4K7 and 22 *μ*F, respectively, in order to get a cut-off frequency of 1.5 Hz. Finally, the signal is amplified and further low-pass filtered by *U*_1_*_B_*. An output offset of 2.5 V is added to obtain a final output voltage range from 0 to 3.0 V, using the gain set by *R*_4_ *=* 5*K*49 and *R*_2_ *=* 1*K*02. *C*_1_ and *C*_2_ are decoupling capacitors to prevent noise from the power supply from entering the signal. *V_OUT_* is the input to the ADC.

### 2.3. Power Source

TherCom runs off batteries. Batteries can be rechargeable or disposable. Among all battery types, we chose NiMH for the following reasons:

- They deliver much closer to their rated capacity. Battery life is defined for 2,700 mAh rechargeable batteries, although current capacities, especially for disposable batteries, are even higher.
- They stay at about 1.2 V for most of their discharge cycle, high enough to feed most of the current available the step-up DC-DC converters.
- They are less expensive than alkaline or Li-ion batteries.
- Chargers are readily available. TherCom includes a Mini-USB connector and a charging circuit for in-place battery recharging from USB power [23].
- They can be recharged hundreds of times (minimum 500 cycles guaranteed).
- Off-the-shelf parts. Potential users are very familiar with this type of batteries.
- Can be recharged indefinitely at low current rates (lower than 120 mA) without damaging the batteries.

**Figure 3.**
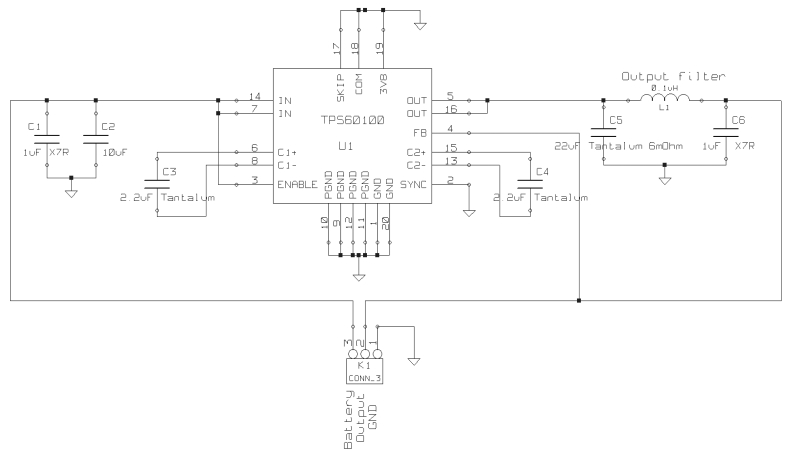
Schematic of the device power source. The core of this block is a step-up DC-DC converter TPS60100.

Two NiMH batteries are used in series to provide an output voltage of approximately 2.5 V. Since the TherCom parts require a higher and stable voltage of 3.3 V, a regulator is needed to boost and keep the voltage constant. The circuit for this task is shown in Figure 3.

We chose the step-up charge pump TPS60100. It generates 3.3 V *±*4% from the battery input, and provides a maximum output current of 200 mA. This is sufficient to power the whole device (maximum current consumption is 60 mA during Bluetooth operation) with an efficiency up to 90%. This DC-to-DC converter does not require any external inductor, only four capacitors. Ceramic capacitors are recommended for their low equivalent series resistance, that enables them to be charged and discharged very fast and therefore filter spikes. An additional output filter (Figure 3) is included to further reduce the ripple.

Power consumption reduction is also very important in portable devices. We applied a number of techniques to minimize the power consumption [24]:

- Switch off external circuits when not needed. I/O pins are used to power the thermistor amplifier and the voltage references.
- The microcontroller periodically goes to sleep and the watchdog timer awakes it for temperature sampling.
- Clock the microcontroller at a lower frequency.
- Configure unused pin ports as outputs.

### 2.4. Voltage References

Measurement accuracy greatly depends on the accuracy of the voltage references. They are used to control the thermistor current source and set voltage offset of the amplifier (Section 2.2.). They are also used to set the full scale of the analog-to-digital converter. Any error in the reference voltages will adversely affect linearity, gain, and range of the data conversion.

This application demands high accuracy and therefore external precision voltage references are required. There are many parts available for this task with different degrees of precision, initial accuracy, long term stability, and temperature coefficient. We chose parts with an error less than 0.05%, and three different voltage levels: 1.225 V (LM4051-1.2) for the thermistor constant current source, 2.50 V (LM4120-2.5) for the thermistor amplifier offset voltage, and 3.00 V (LM4120-3.0) for setting the full-scale of the analog to digital converter.

### 2.5. CPU

The CPU of the device is based on the Microchip PIC18F8722 microcontrollers family. The main features of this family are:

- High performance 8 bit RISC CPU.
- 40 MHz/10 MIPs sustained operation.
- 2.0 to 5.5 V operation.
- Flash, SRAM and EEPROM memory. One of the restrictions in embedded systems is the amount of memory available. This memory shortage is usually overcome by using additional external memories (such as microSD cards). However, body temperature measurement does not require high sampling rates and therefore small memories suffice to store all the data for long periods.
- Digital and analog ports. Digital ports are used to control external LEDs, power the amplifiers and the voltage references. Analog ports are used to acquire thermistor voltage, input ADC voltage references, and monitor batteries level.
- 4 timers.
- Up to 25 mA sink/source pin capability. Digital outputs power the amplifiers during sampling and the rest of the time remain in high impedance to save battery power. This sink/source capability is sufficient for the amplifiers and voltage references.
- Up to 16 analog channels. 2 analog channels are used for each thermistor, 2 channels as the ADC voltage references, 1 channel to monitor battery level, and another channel to monitor recharging block. Devices with 12 and 16 ADC channels are available in this family. Resolution of the converter is 10 bits, and the measurement range is 10° C. That gives a resolution of 0.01 °C per count, better than the accuracy of the sensors. Sample and hold circuit is included in the ADC.
- Up to 25 Ksps.
- Full-duplex asynchronous or half-duplex synchronous addressable USART. Serial interface is employed to communicate with the Bluetooth module.
- Programmable low voltage detect.
- Watchdog timer.
- In-System Programming Method.

### 2.6. ADC

Analog to digital conversion is performed by the converter embedded in the microcontroller. The resolution is 10 bits, with 1,023 counts for the maximum level. There is only one analog-to-digital converter on the microcontroller, namely, only one channel can be converted at a time. An analog multiplexer connects the selected input to the holding capacitor and to the ADC.

The input range is set by high and low voltage references. Although VDD or other internal references can be used, the high voltage reference was taken from the precision voltage reference of 3.0 V described in Section 2.4. for more accuracy and stability.

The ADC is of the successive approximation type. The steps to perform a conversion are:

- Configure pin as analog input.
- Select channel.
- Enable ADC module.
- Start conversion.
- Wait for the conversion to be completed.
- Read the result.

These steps are carried out by the firmware, at a sampling rate of one sample per second.

### 2.7. Bluetooth Module

Wireline data transmission is very inconvenient in mobile clinical applications. To overcome this limitation, we included Bluetooth interface for wireless data transmission. Additionaly, Bluetooth specifications can be supported by a number of different hardware platforms such as cell phones, laptop computers, personal digital assistants and embedded devices. Modules containing the radio hardware are provided by a variety of vendors, and often driver software is also included in order to facilitate design and integration tasks, avoiding, for example, the need for antenna design or RF certification. They virtually eliminate the need to have specific Bluetooth knowledge and simplify programming.

The Bluetooth module chosen for the proposed system is a Linkmatik module [25]. It provides a full duplex point-to-point connection similar to that of a standard serial cable, at a transfer rate of up to 50 Kb/s. It can work in slave mode, waiting for a remote device to connect after initialization, master mode, looking for specific devices to connect to, and even connect to other Linkmatik module. We programmed the module to work in slave mode, the host computer connects to the TherCom device for offline or real time data download. Up to 7 devices can be connected in real time simultaneously to the host computer. It is possible to set different security levels, only devices that present the correct password are permitted to connect to the host.

The distance communication range is 100 m. It can be powered with a voltage between 3 V and 5 V. The LinkMatik Bluetooth module is FCC/CE/IC compliant and does not need re-certification if the integral antenna is used.

### 2.8. Firmware

The microcontroller's firmware carries out the following tasks:

- Bluetooth module control. A high priority periodical interrupt polls module to read commands from the host computer (set device name, start acquisition, configure mode, get battery level, set date, set time, and retrieve data from memory). It also sends data to the host computer with the format: Date (yyyy-mm-dd), time (hh:mm:ss) and the two temperature samples (float values with two decimal digits). All the fields are separated by tabs. Temperatures are computed according to preprogrammed calibration curves.
- Off-line data acquisition in Holter mode. Bluetooth module is switched off to save power and enable the device to function for up to 10 days. A low priority interrupt samples the two channels every second. The device is in sleep mode for 90% of the time. It is awaken 10 ms before ADC inputs are sampled to get stable values. When 60 samples have been acquired, the mean is computed and stored in EEPROM with the format described above. All data are downloaded to the host computer afterwards, once the recording ends by restarting the device.
- Real time data acquisition. Functionality is similar to that of Holter mode, except that the Bluetooth module is not switched off, and when the mean is computed, resulting data are both stored in memory and transmitted to the host computer. Storing data in memory in real time mode enables the device to download lost data in case communication between host computer and TherCom was interrupted (for example, because of device out of range due to monitored patient displacement).
- Power control. In order to save battery power, peripherals are switched off during most of the time. They are only active when a temperature sample is acquired. Additionally, microcontroller is in sleep mode until watchdog timer periodically throws an interrupt to awake it. Then, one digital output is set active to power the thermistor amplifier and the voltage references. Both temperature channels are then measured, peripherals are switched off, and the microcontroller enters sleep mode again until next cycle. In offline mode, the Bluetooth module is also disabled.

In addition, firmware implements a real time clock (RTC) based on an internal temporization of 1s and a low priority interrupt. Current date and time are set every time a new recording is started, either in real time or offline. It provides seconds, minutes, hours, day, month and year information that are associated to each pair of temperature values.

### 2.9. Physical Features

The device dimensions are 90 *×* 63 *×* 28 mm and weights 250 g—most of it due to the two AA batteries. The maximum current consumption is 60 mA during Bluetooth discovery and connection. In real time mode, current consumption is around 40 mA, whereas it is lower than 4 mA in Holter mode.

To protect the device against X-Ray, usual practice in many monitored inpatients, electronic parts are covered by a special fabric. The fabric is a jacquard one, of 210 *g/m*^2^. It is made out of 100% cotton. The fabric was chemically bleached. Some metals have been used with polyurethane for coating it on one face. Afterwards the fabric was dried with a dry machine by IR made by Serigrafía S.L. kind TD-20, 220 V.

### 2.10. Calibration

For accurate temperature measurements, it is possible to calibrate the device with high precision resistors. We chose the following reference resistance points within the temperature measurement interval: 1K21, 1K24, 1K30, 1K37, 1K43, 1K54, 1K65, and 1K74 (0.1% tolerance). Calibration points are set for each channel at the host computer, and they are permanently stored in the device.

### 2.11. Software Application

TherCom includes a software application for the host computer. This application enables the user to configure a TherCom device, start a recording, off-line data download, or calibrate the device. Additionally, it contains standard time-series analysis capabilities and the ability to compute the approximate entropy and sample entropy of the temperature recordings. These signal complexity calculations contribute to capture the dynamic aspects of temperature regulation. Some results in this regard have already been published, see [12].

### 2.12. Tools Used

Schematics and PCBs where designed using the Kicad electronic suite. PC programming was developed using the Eclipse platform and C++. All the graphic user interfaces were programmed using wxWidgets to enable crossplatform capabilities.

## 3. Results and Discussion

We conducted a Mann-Whitney U test to assure that both channels measure equally. In this type of temperature monitor it is critical for both channels to provide identical high-accuracy temperature readings. We used the calibration precision resistors as the items measured in this test, and 10 equal resistors were measured randomly 3 times.

The results of the statistical tests confirmed the absence of statistically significant differences between the channels *(α =* 0.01). For the non-parametric test, MW statistic was *U =* 388.5, with a*p-*value = 0.3164. Therefore, it did not reject the hypothesis of equal means *H*_0_. An additional normality test (Shapiro-Wilks) provided the following results: *ω_A_=* 0.8351, *p*–value = 0.00019, *ω_B_=* 0.7973, *p*–value = 0.00003, and therefore normality was not accepted. It confirmed that the non-parametric test MW had been used correctly. From these tests, we can conclude that both channels measure in the same way.

In order to assure that the device was properly calibrated and functioning correctly, we compared its performance with a reference digital precision thermometer (Testo 110). In this experiment, the temperature of hot water was measured at 60 s intervals using both devices. In total, 50 measures were taken for each one. Then, we carried out three hypothesis tests to determine if both devices could be considered to measure equally. First, a t-test was conducted to compare the averages of the measurements [26]. The results obtained were *t* = −0.0550252 with p-value = 0.956228, which implied that both averages could be considered equal. Then, an F test [26] was used to find out if the variability was the same in both devices. The results in this case were F = 1.12067 with p-value = 0.688653, confirming the initial hypothesis. Finally, a non parametric Kolmogorov-Smirnov test [26] also confirmed that both exhibit the same statistical distribution, since K-S = 0.297044 with p-value = 0.999993. Therefore, we concluded that the accuracy of TherCom was equal to that of Testo 110, namely, *±* 0.2 *^°^*C. The specifications of TherCom are shown in Table 1.

This device has several functions, not met by other, to our knowledge:

- It provides a real-time, wireless monitorization of temperature. This may be useful both in patients admitted to a critical care unit and in relatively independent patients admitted to a general ward (and able to move freely).
- It provides two channels, thus allowing for a central and a peripheral temperature reading. This offers a good insight both on the thermoregulatory system and on the general perfusion status. Arguably, measuring the central/peripheral gradient will allow for an early detection of an impending haemodynamic deterioration or bacteraemia.
- Recording the lectures on a memory allows for its use as a temperature Holter device. This may be extremely useful in patients attending an outpatient clinic because of occasional fever bouts, not confirmed during the physical exam.
- It provides a complexity analysis of the temperature profile. ApEn has proved to have prognostic implications on survival of critical patients (reference), and could also be useful in the differential diagnosis of certain diseases.

As for the placement of sensors, in critically ill patients the central temperature is usually measured through an indwelling bladder catheter with a thermistor (i.e., Mon-a-therm Foley Temp). In free dwelling patients, a thermistor sensor is placed in the external auricular channel (i.e., Mon-a-therm Tympanic probe) and sealed from the exterior. Other locations (esophageal, rectal, etc.) may also be used. The peripheral sensor is placed in the skin. Depending on the experimental protocol, it may be located in the thorax or more peripherally (wrist, ankle).

This device was tested in a sample of 30 free dwelling healthy volunteers 20 to 70 years old . Subjects were monitored for 24 hours while following their normal life. Temperatures were recorded every minute at the external auditory channel (EAC) and on the skin (intersection of the 5th intercostal space and the anterior axillary line). A Cosinor analysis and Approximate Entropy (ApEn)(*m* = 2, *r* = 0.15sd, *N* = 180) were calculated for both temperatures. Median temperature was 35.55 °C (interquartile range (IR) 0.77 in EAC and 36.62 *^°^*C (IR 1.61) in the specified skin location. Median gradient between EAC and skin was 0.93 (IR 1.57). A circadian rhythm was present both in EAC and skin temperature, with a mean amplitude of 0.44 °C and an acrophase at 21:02 for EAC and 0.70 °C and 00:42 for the skin. During the night there was a sizable increase in peripheral temperature, with a decrease in gradient and a loss of complexity in the temperature profile, most significantly in the peripheral temperature. A detailed clinical evaluation can be found in [27].

## 4. Conclusion

We described a portable precision body temperature monitor. The temperature monitor has two inputs in order to be able to read two sensors simultaneously with an accuracy of *±*0.2 °C and a resolution of 0.01 °C. The TherCom monitor can log up to 1500 readings with date and time stamps. The logging interval is 60 seconds, although internal readings are acquired every second for averaging. The batteries can provide 50h of continuous operation in real time mode or more than 10 days in ambulatory mode. We report the results of our characterization study, test study, reproducibility and reliability study, and clinical study. The device clinical validation was carried out at Mostoles Hospital in Madrid (Spain). Patients admitted to the ICU were monitored in real time mode and patients at the internal medicine unit were monitored in Holter mode. Healthy subjects were also monitored in Holter mode for reference purposes.

The objective of this paper is to provide an accurate and thorough description in order to ensure the research community can reproduce the design. The temperature device we proposed in this paper has been designed to capture the dynamic aspects of temperature in order to enable researchers to conduct further studies in the area of temperature regulation, thermal physiology, and clinical thermometry. Additionally, it is intended to serve as a clinical device to enable physicians to implement current advances in thermal physiology as part of their day-to-day clinical practice. Specifically, in addition to serve as a research tool, the proposed device has been designed to address common problems encountered in the clinical practice.

## Figures and Tables

**Figure 4.**
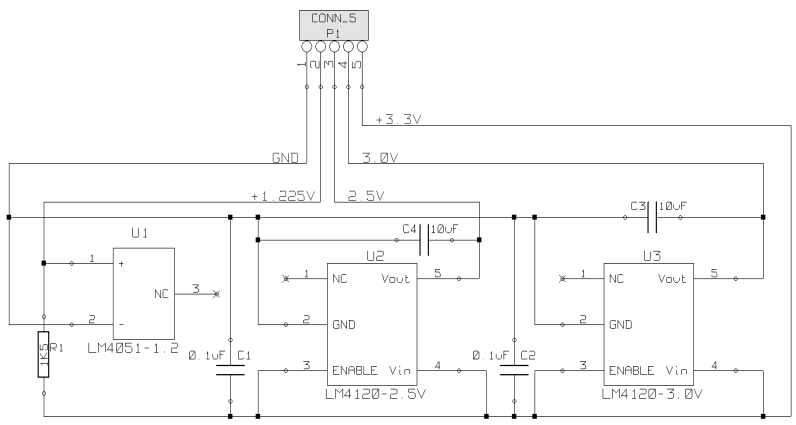
Circuit schematic of the voltage references.

**Figure 5.**
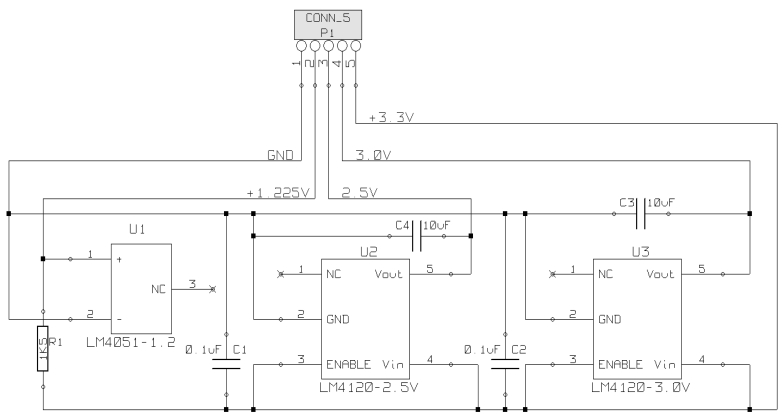
TherCom device. In its final form, on the left, two sensors are attached to the device, for timpanic and peripheral temperature monitoring. The opened device on the right shows the battery holder, Bluetooth module, the two amplifiers, and the microcontroller (the rest of the parts are not visible).

**Table 1.** Results of the Characterization Study and Final TherCom Specifications.

Specification	Result
Inputs	2 channels thermistor
Temperature range	31 °C to 41 °C
Characterizations	Steinhart-Hart, YSI-400
Temperature accuracy	*±* 0.2 °C
Temperature resolution	0.01 °C
Measurement interval	1 minute
Data logging	Up to 10 days
Averaging	60 samples/minute
Probe connection	Universal 2-way male connector
Communications	Bluetooth interface
Battery	2xNiMH AA rechargeable
Size	90 *×*63 *×* 28*mm*
Weight	250 g
